# Phenotypic flexibility as a measure of health: the optimal nutritional stress response test

**DOI:** 10.1007/s12263-015-0459-1

**Published:** 2015-04-21

**Authors:** Johanna H. M. Stroeve, Herman van Wietmarschen, Bas H. A. Kremer, Ben van Ommen, Suzan Wopereis

**Affiliations:** TNO, PO Box 360, 3700 AJ Zeist, The Netherlands

**Keywords:** Phenotypic flexibility, Metabolic challenge, OGTT, Metabolic health, Nutritional stress response test

## Abstract

Nutrition research is struggling to demonstrate beneficial health effects, since nutritional effects are often subtle and long term. Health has been redefined as the ability of our body to cope with daily-life challenges. Physiology acts as a well-orchestrated machinery to adapt to the continuously changing environment. We term this adaptive capacity “phenotypic flexibility.” The phenotypic flexibility concept implies that health can be measured by the ability to adapt to conditions of temporary stress, such as physical exercise, infections or mental stress, in a healthy manner. This may offer a more sensitive way to assess changes in health status of healthy subjects. Here, we performed a systematic review of 61 studies applying different nutritional stress tests to quantify health and nutritional health effects, with the objective to define an optimal nutritional stress test that has the potential to be adopted as the golden standard in nutrition research. To acknowledge the multi-target role of nutrition, a relevant subset of 50 processes that govern optimal health, with high relevance to diet, was used to define phenotypic flexibility. Subsequently, we assessed the response of biomarkers related to this subset of processes to the different challenge tests. Based on the obtained insights, we propose a nutritional stress test composed of a high-fat, high-caloric drink, containing 60 g palm olein, 75 g glucose and 20 g dairy protein in a total volume of 400 ml. The use of such a standardized nutritional challenge test in intervention studies is expected to demonstrate subtle improvements of phenotypic flexibility, thereby enabling substantiation of nutritional health effects.

**Electronic supplementary material:**

The online version of this article (doi:10.1007/s12263-015-0459-1) contains supplementary material, which is available to authorized users.

## Introduction

Health as formulated by WHO in 1948, is defined as a condition of complete physical, mental and social well-being and not merely the absence of disease or infirmity (WHO 2006). Diet plays an essential role in maintaining health. However, providing scientific proof of the relationship between diet and dietary components and health has proven to be difficult. This is partly due to the subtleties of the effects of diet on health, but a more fundamental cause lies in the design of studies and the biomarkers used to determine health effects. Currently, substantiation is based on demonstrating that diet or dietary ingredients prevent specific diseases by reducing disease-risk biomarkers or surrogate endpoints derived from dietary intervention studies and epidemiological studies. There is a growing awareness that health includes adaptation and flexibility to continuously changing environmental conditions. Health has been redefined (Huber et al. 2011) along the lines of flexibility, adaptability, elasticity, robustness, and others. In the context of metabolic health, we term this ability to adapt “phenotypic flexibility” (van Ommen et al. 2014). Consequently, the quantification of the stress response reaction should be informative about the health status.

**Fig. 1:**
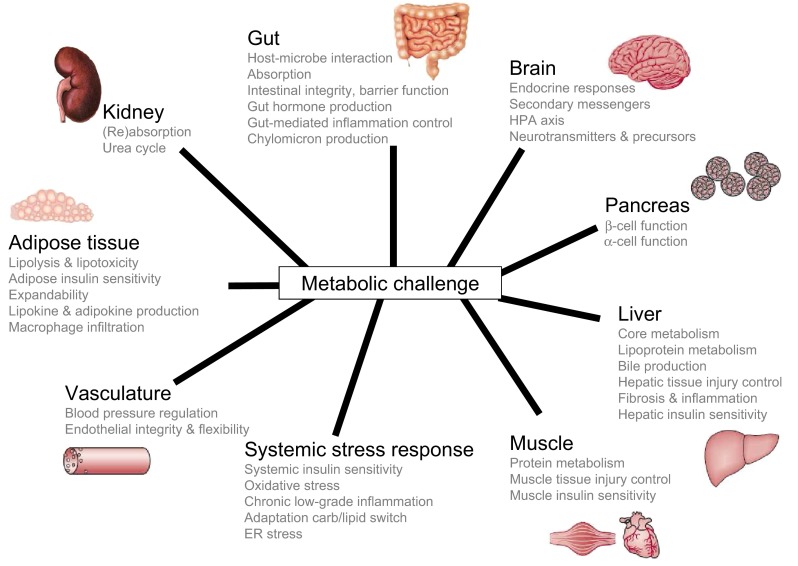
Physiological processes involved in phenotypic flexibility. Thirty-five different physiological processes that may be influenced by food and nutrition have been defined. The optimal nutritional stress challenge should trigger all these physiological processes, so that it allows the broad quantification of nutritional health effects

**Fig. 2:**
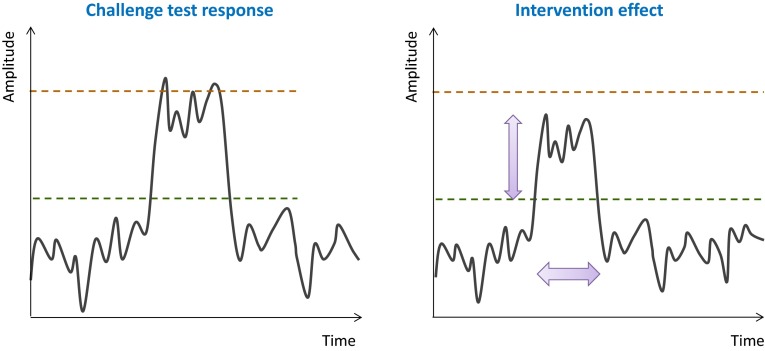
Graphical representation of a single-marker response profile during homeostasis and upon challenge test before and after intervention. The challenge test evokes a response in concentration of a biomarker that is representative of a physiological system of interest which returns to homeostatic levels after a period of time. The intervention should ideally lead to an improved challenge response in terms of amplitude and duration

Appreciating the importance of maintaining flexibility as a key component of optimal health opens new avenues toward “biomarkers of health” rather than “biomarkers of disease,” which are urgently needed in nutrition research. Most nutrients, when consumed in appropriate amounts, compositions, time of day, season, etc., play key roles in the well-orchestrated machinery maintaining phenotypic flexibility (van Ommen et al. 2014). Certain dietary components, by excess or by lack of, impair phenotypic flexibility (van Ommen et al. 2014). To be able to measure a person’s phenotypic flexibility, the homeostasis of that person must be perturbed, followed by determining the response of single or multiple markers during a limited period of time. Figure 1 gives an overview of the main physiological processes in the context of diet-related health. A detailed description of these processes in relation to metabolic health is considered out of scope for this review. By careful design of a nutritional stress response test that triggers most of the physiological processes involved in diet-related health, the effect of food and food products on phenotypic flexibility can be quantified. Ideally, each of these processes can be quantified by determining the timeline of stress response of specific parameters in blood samples (Online Resource 1). These markers can be used to discover intervention-induced changes in the duration and degree of the flexibility of the quantified physiological systems as compared to control or placebo intervention, for instance to substantiate health effects of foods (Fig. 2). Such findings can possibly also be related to various states of impaired health and ultimately may predict the development of certain diseases in the future.

Challenge tests based on carbohydrates, lipids, proteins and/or combinations thereof have been designed to temporarily disturb the body’s homeostasis. In response to such dietary stressors, the system will strive to restore homeostasis, usually within hours. The extent of the disruption and the speed of recovery to homeostasis are health indicators. The development of a challenge test that is sufficiently sensitive to measure subtle changes due to food interventions is a key step toward “biomarkers of health.” In this context, a large body of literature has been published using different metabolic challenge tests to assess health status and intervention effects. Over the years, specific metabolic challenge tests have been developed to elicit a strong response in the body in a relatively short period of time with different sets of physiological processes responding to these different challenge tests. The most widely used challenge test is the oral glucose tolerance test (OGTT), which specifically aims to establish the functional flexibility of the glucose metabolism system. Although the OGTT can be considered to be the most standardized challenge test, it is less representative for an individual’s diet, and therefore, the effects may not include the total orchestra of physiological systems representative for phenotypic flexibility. Additionally, lipid tolerance tests, protein tolerance tests and challenge tests based on a mixture of these macronutrients have been developed to elicit responses in different sets of processes. Closer to real life, challenge meals such as a Mediterranean or vegan diet are studied, which could be considered as the least standardized challenge tests.

The purpose of this review is to provide an overview of findings with the different challenge tests and to extract insights on composition-based differences with respect to processes triggered, observed differences between healthy and sub-healthy people, including subjects with obesity, metabolic syndrome (MetS) and prediabetics, and the ability to modify responses by nutritional interventions. These insights were used to guide the design of an optimized nutritional stress test that has the potential to be adopted as the golden standard in nutritional health research.

For the current paper, peer-reviewed scientific literature was collected from PubMed with the following criteria: (1) the papers should describe human studies in which a metabolic challenge test is conducted. These challenge tests could consist of pure glucose, lipid or protein, as well as combinations thereof or as complete meals; (2) the papers should include objective measurements of the response to the challenges, such as plasma metabolites, proteins, genes and physiological responses such as heart rate variability.

In total 61 papers were retrieved that fit the criteria. OGTT is discussed in 17 of those papers, the oral lipid tolerance test (OLTT) in 19 papers, the oral protein tolerance test (OPTT) in six papers and combined challenge tests (carbohydrates, lipids and protein, OPGLTT) are discussed in 17 papers. The review is organized in sections describing the responses to a challenge test in healthy subjects, followed by a section describing the responses in subjects with impaired health conditions. After the molecular characterization of OGTT, OLTT, OPTT and OPGLTT, special attention is given to the relationships between macronutrient composition and types of fat in the challenge tests and the biomarker responses. This section is followed by a discussion of the effects of nutritional interventions on challenge test responses. The review finishes with some remaining discussion topics and conclusions. A full list of the papers and specifications is given as supplementary material (Online Resource 2). Furthermore, the different physiological processes from Fig. 1 and the different biomarkers that were defined as a read-out for these processes have been given in Online Resource 1.

### The oral glucose tolerance test

The best-known challenge test is the OGTT, as the mainstay for diagnosing diabetes for decades. It is based on the principle that patients with diabetes have more difficulty clearing their blood glucose after a glucose bolus (75 grams after overnight fasting) in comparison with healthy subjects. An OGTT assesses diabetes more efficiently than fasting plasma glucose as it quantifies altered postprandial metabolism (Bartoli et al. 2011). In total 17 papers were reviewed presenting metabolic effects of an OGTT, 13 of those in healthy subjects and nine in various groups with sub-optimal health status. Table 1 gives an overview of the physiological processes that have been studied in healthy and sub-optimal healthy individuals.

**Table 1:** Processes of phenotypic flexibility modulated by OGTT

Organ system	Process	Direction (↑, ↓, –)	Disturbed
Gut	Incretin production	(GLP-1) ↑	
Adipose tissue	Lipolysis	↓	IGT
	Adipokine production	–	
Systemic stress	Insulin sensitivity	↑	MetS, IGT, IFG, others
	Oxidative stress	↓	MetS
	Nitrosative stress	↑	T2DM
	Inflammation	–	
	ER stress	↓	
Muscle	Proteolysis	↑	IFG
	Muscle injury control	↓	
Liver	β-oxidation	↓	IGT
	Ketogenesis	↓	IGT
	Glycolysis/Gluconeogenesis	↑	IGT
	Purine degradation	↓	
	Lipoprotein production	↓	MetS, IGT
	Bile production	↓	IGT
Kidney	Urea cycle	↓	
Vasculature	Blood pressure regulation	↓	MetS
	NO formation	↓	
	Adhesion	Subtle ↑	T2DM
Pancreas	Insulin secretion	↑	MetS
Brain	HPA axis	–	MetS
	Neurotransmitters and precursors	↓	

*MetS* metabolic syndrome, *T2DM* type 2 diabetes mellitus, *IFG* impaired fasting glucose, *IGT* impaired glucose tolerance, *ER stress* endoplasmic reticulum stress

Glucose homeostasis is a complex physiological process involving the orchestration of multiple organ systems. Glucose ingestion after an overnight fast triggers an insulin-dependent, homeostatic program along four key metabolic axes: lipolysis, ketogenesis, proteolysis and glycolysis, reflecting a switch from catabolism to anabolism (Shaham et al. 2008). With respect to lipolysis, triacylglycerol (TG) lysis in adipose tissue is inhibited leading to a reported 50 % reduction in glycerol and a 90 % reduction in total free fatty acids (FFA) 2 h after glucose intake. A compositional change in FFA has been reported as well (Shaham et al. 2008; Skurk et al. 2011). Unsaturated fatty acids such as oleic acid and linoleic acid were suppressed faster and stronger than saturated fatty acids such as stearic acid (Spégel et al. 2010). The ratio of unsaturated-to-saturated FFA species was found to decrease by 79 % during the OGTT (Zhao et al. 2009). Furthermore, the long-chain polyunsaturated fatty acid (PUFA) levels decreased less strongly, indicating a relative abundance of PUFAs compared to the saturated and monounsaturated fatty acids, which was reflected in the composition of newly formed very low-density lipoprotein (VLDL) (Zhao et al. 2009). Plasma oxylipin derivatives, which are lipid mediators involved in inflammation and cellular growth processes and synthesized from fatty acids arachidonic acid, linoleic acid, eicosapentaenoic acid and docosahexaenoic acid, are significantly reduced 2 h after OGTT, which is a similar response as for their FFA precursors (Wopereis et al. 2013). FFA will be taken up by the liver and re-esterified into TG, released by lipoproteins. An OGTT has been shown to result in a number of significant changes in the lipoprotein production of 10 healthy volunteers (Ogita et al. 2008). TG, remnant-like particles-cholesterol (RLPC) and remnant-like particles-triglyceride (RLPTG) levels in serum gradually decreased upon glucose intake reaching a significantly lower level after 2 h. In contrast, Ceriello et al. (2004) did not show this decrease in TG levels upon OGTT. These conflicting results may be explained by the heterogeneity of the response of TGs to an OGTT as found in 378 subjects from the Framingham Heart Study Cohort. TGs of lower carbon number and double-bond content decreased 2 h after glucose intake, while TGs of higher carbon number and double-bond content increased (Rhee et al. 2011).

Hepatic synthesis of ketone bodies, i.e., ketogenesis, is inhibited, leading to halved β-hydroxybutyrate levels 2 h after an OGTT (Shaham et al. 2008; Skurk et al. 2011). Similarly, other intermediates of β-oxidation, i.e., C8:0-carnitines, C10:0-carnitines, C12:0-carnitines, and C14:1-carnitines, decreased 60–70 % upon glucose intake (Zhao et al. 2009). The switch from proteolysis in skeletal muscle and associated release of amino acids to amino acid uptake and protein synthesis is shown by decreased plasma concentrations of several amino acids and urea cycle intermediates in response to OGTT (Shaham et al. 2008; Skurk et al. 2011; Wopereis et al. 2009). Glycolysis is stimulated leading to increased pyruvate, lactate and alanine levels between 30 and 60 min after the glucose intake (Shaham et al. 2008).

There is a clear connection between glucose intake and incretin production by the gut, such as glucagon-like peptide (GLP)-1. Upon OGTT, the GLP-1 plasma levels increased already after 15 min and remained stable up to 180 min (Skurk et al. 2011). Also processes less well known to be linked to glucose metabolism have been reported to respond upon OGTT. Bile acid synthesis is decreased upon OGTT, as reflected by decreased plasma levels of 7α-hydroxy-4-cholesten-3-one (Matysik et al. 2011), and unconjugated bile acids are converted to forms conjugated glycine or taurine (Matysik et al. 2011; Shaham et al. 2008; Zhao et al. 2009). Other processes related to systemic stress, such as inflammatory and oxidative stress responses, have also been shown to be triggered by the OGTT. The OGTT has been shown to induce a small temporary increase in circulating leukocytes, suggesting an inflammatory response (Van Oostrom et al. 2003; Wopereis et al. 2013). However, a comparison of the OGTT challenge responses to a water control challenge in fasted healthy adults revealed that no OGTT-specific postprandial changes were induced as evaluated by a number of classical pro-inflammatory markers, i.e., Interleukin (IL)-1β, IL-6, IL-8, IL-10, IL-12p70, tumor necrosis factor (TNF)-α and interferon (IFN)-γ (Wopereis et al. 2013). The increase in leukocytes was primarily due to the increased numbers of neutrophils (Van Oostrom et al. 2003; Wopereis et al. 2013), which have been linked to changes in endothelial function. This is also reflected by subtle increases in the endothelial adhesion markers intracellular adhesion molecule (ICAM)-1, vascular cellular adhesion molecule (sVCAM)-1 and sE-selectin (Ceriello et al. 2004; Wopereis et al. 2013). The OGTT reduces several markers of oxidative stress, such as uric acid, thiobarbituric acid-reactive substances (TBARS) and metabolic intermediates of the glutathione synthesis pathway (Nakatsuji et al. 2010; Wopereis et al. 2009). This decrease in oxidative stress might be due to the decrease in TG levels and blood pressure upon OGTT, which have been known to correlate with oxidative stress levels (Bae et al. 2001). In contrast, levels of nitrotyrosine, a marker of peroxynitrite and nitrosative stress generation, and also of advanced glycation end products (AGEs), a heterogeneous group of molecules formed from the non-enzymatic reaction of reducing sugars with free amino groups of proteins, lipids, and nucleic acids by the high glucose load, were found to increase in response to OGTT (Ceriello et al. 2004; Williams et al. 2012).

### OGTT response in sub-healthy and diseased conditions

Subjects with sub-optimal health status have been reported to show differential OGTT responses for several processes related to phenotypic flexibility. In addition to dysregulated glucose and insulin responses, triglyceride, blood pressure and oxidative stress responses to OGTT in men with abdominal obesity (with waist circumference (WC) >85 cm) were blunted in comparison with men with WC <85 cm. (Nakatsuji et al. 2010). Metabolic markers related to proteolysis (branched chain amino acids and methionine), lipolysis (glycerol), ketogenesis (β-hydroxybutyrate) and glycolysis (lactate) showed blunted responses to OGTT in subjects with impaired glucose tolerance (Shaham et al. 2008). Increases in plasma concentrations of the bile acid glycochenodeoxycholic acid were reduced in response to OGTT in these subjects (Shaham et al. 2008), which is in line with a significantly decreased chenodeoxycholic acid pool in type 2 diabetes mellitus (T2DM) patients (Brufau et al. 2010). Interestingly, Shaham et al. (2008) showed that 2 h metabolite OGTT change of leucine/isoleucine and glycerol is highly correlated to the elevated fasting insulin levels of these subjects with impaired glucose tolerance. It was demonstrated that some individuals with high fasting insulin exhibit a blunted decline in lipolysis (glycerol), whereas others exhibit a blunted decline in proteolysis (leucine/isoleucine). These findings suggest different origins for the high insulin levels in these subjects. Individuals with insulin resistance had a blunted decrease in TGs with lower carbon number and double-bond content during OGTT (Rhee et al. 2011). Type 2 diabetes mellitus (T2DM) patients showed prolonged OGTT-induced increases in endothelial function markers, sICAM-1, sVCAM-1 and sE-selectin, compared to healthy subjects (Ceriello et al. 2004). A similar difference was observed for the oxidative stress marker nitrotyrosine (Ceriello et al. 2004). An exaggerated glucose response was observed in people with a major depressive disorder (Garcia-Rizo et al. 2013) and in people with a subtype of schizophrenia (Kirkpatrick et al. 2009), which is in line with the association of hypothalamic pituitary adrenal (HPA) axis activity with an altered glucose response upon an OGTT (Weber-Hamann et al. 2005).

### The oral lipid tolerance test (OLTT)

In 1907, Neumann was one of the first to apply an oral fat challenge test to study the dynamics of chylomicrons in blood (Neumann 1907). Nowadays, challenge tests composed of lipids are used to monitor fatty acid β-oxidation. Various types of lipid challenges have been used in scientific research to answer a diverse set of research questions. A total of 19 papers were reviewed presenting metabolic effects of an OLTT according to the above-mentioned criteria. Six of these papers report on studies that used a 100 % lipid challenge test. Thirteen other studies made use of a challenge test predominantly consisting of lipid (over 85 E%) which are included in this section as well. Fifteen papers described the response to an OLTT in healthy subjects and 15 papers described the response to an OLTT in various groups with an impaired health status. The phenotypic flexibility responses are summarized in Table 2. As differential results may depend on the macronutrient composition of the challenge, this review will address this aspect in a dedicated paragraph.

**Table 2:** Processes of phenotypic flexibility modulated by OLTT

Organ system	Process	Direction (↑, ↓, –)	Disturbed
Gut	Incretin production	(GIP) ↑ (GLP-1) **↓**	NASH, MetS
	Gut-mediated inflammation	–	Obesity, T2DM, IGT
Adipose tissue	Lipolysis	↑	Obesity, MetS, NASH
	Adipokine production	↑	NASH, obesity
Systemic stress	Insulin sensitivity	–	
	Oxidative stress	↑	NASH, obesity
	Nitrosative stress	↑	T2DM
	Inflammation	–	MetS
	Metabolic flexibility	↑	MetS, obesity
Liver	Lipoprotein production	↑	NASH, T2DM, obesity, IGT
	Hepatic injury control	↑	NASH
Vasculature	Blood pressure regulation	↓	Obesity
	Adhesion	↑	T2DM
	Vascular flexibility	↓	T2DM
Pancreas	Insulin secretion	↑^a^	MetS, obesity

*MetS* metabolic syndrome, *T2DM* type 2 diabetes mellitus, *IFG* impaired fasting glucose, *IGT* impaired glucose tolerance, *NASH* non-alcoholic steatohepatitis

^a^Dependent on carbohydrates in OLTT

Lipids ingested after an overnight fast are digested and absorbed in the gut, and enter the blood stream packaged into chylomicrons, resulting in increased VLDL subfraction responses of intestinal origin (Musso et al. 2009). The chylomicron remnants are taken up by the liver and the residual lipids are secreted and transported in the blood via VLDL particles. This is reflected by increased plasma concentrations of VLDL (Musso et al. 2009) and TGs with maximum levels 4 h after OLTT (Musso et al. 2009; Wopereis et al. 2013). As early as 1916, the first observations of stable plasma total cholesterol levels after a fat load were reported (Blix 1925; Bloor 1916) as well as observations of slight increases of total cholesterol levels (Brun 1940; Hiller et al. 1924; Umpaichitra et al. 2004).

Similar to ingestion of glucose, OLTT triggers the secretion of C-peptide and insulin (Blaak et al. 2006; Esser et al. 2013a, b, 2014; Umpaichitra et al. 2004; Wopereis et al. 2013; Wuesten et al. 2005), which may be explained by the small amounts of carbohydrate present in the OLTT that were not 100 % based on lipids. This insulin response was still significantly different from baseline values 3 h after intake of OLTT (Esser et al. 2013a, b). However, this insulin response is less pronounced as compared to OGTT (Wopereis et al. 2013). Insulin inhibits the process of lipolysis, resulting in decreased plasma concentrations of FFA 1 and 2 h after fat intake (Blaak et al. 2006; Esser et al. 2013a, b). Increased FFA concentrations have been reported from 4 h onwards after lipid intake (Esser et al. 2013a, b; Musso et al. 2009; Wuesten et al. 2005), suggesting that the process of lipolysis was no longer inhibited. Plasma glucose concentrations observed upon OLTT showed a similar response, decreasing glucose levels at 1 h that subsequently returned to baseline concentrations at 3 h after the challenge (Blaak et al. 2006).

In response to the lipid load, the gut secretes the incretin glucose-dependent insulinotropic polypeptide (GIP), with peak concentrations at 2 h post challenge (Musso et al. 2009). No response was observed for the incretin GLP-1 in healthy subjects (Kardinaal et al. unpublished work). Adipokines resistin and adiponectin increased in response to OLTT (Musso et al. 2009). As expected, fat oxidation increased in response to an OLTT and stabilized 1 h after the OLTT (Blaak et al. 2006).

The endothelial response capacity upon an OLTT has been extensively studied (Ceriello et al. 2004; Esser et al. 2013a, b, 2014; Wopereis et al. 2013). The OLTT is associated with changes in blood pressure and increased heart rate as compared to control challenges, which was observed in both young and middle-aged men (Esser et al. 2013a, b, 2014; Gosmanov et al. 2010). An OLTT elicits a decrease in augmentation index (AIX), which is commonly accepted as a measure of the enhancement (augmentation) of central aortic pressure and represents arterial stiffness. In middle-aged men, OLTT increased plasma concentrations of IL-8, sICAM-1, sICAM-3 and sVCAM-1 and leukocyte adherence marker expression, which was accompanied by a decrease in flow-mediated dilation (FMD), a measure for endothelial function. In young healthy men, the postprandial decrease in FMD was less pronounced, and AIX values were negative, suggesting no augmented central systolic blood pressure (Esser et al. 2013a, b). Together with the absence of a response of plasma sICAM-1, sICAM-3 and sVCAM-1, this suggests that in young healthy men, in contrast to middle-aged men, an OLTT does not adversely affect endothelial function (Esser et al. 2013a, b, 2014). Elevations in endothelial adhesion factor sE-selectin levels were observed to an OLTT (Ceriello et al. 2004), but multiple other studies did not confirm this finding (Esser et al. 2013a, b, 2014; van Dijk et al. 2012; Wopereis et al. 2013). Oxylipins associated with endothelial inflammation and function also responded to OLTT, e.g., LOX-derived 9-HODE and 13-HODE and CYP-derived 11,12-DiHETrE and 14,15-DiHETrE (Strassburg et al. 2014; Wopereis et al. 2013). Interestingly, this postprandial response is very different from their precursors linoleic acid and arachidonic acid, indicating that the OLTT actively modulates endothelial inflammation and vascular function and that it is not simply the normal physiological response to fasting. It has been suggested that CYP-derived DiHETrE’s may be released from vascular endothelium leading to vasodilation, and that LOX-derived oxylipins can be released by lipoprotein lipase on the endothelium leading to increased expression of TNF-α, sVCAM and sE-selectin (Shearer and Newman 2009; Wang et al. 2009).

Also inflammation-related processes in response to an OLTT have been subject of several studies (Esser et al. 2013a, b, 2014; Wopereis et al. 2013). It has been shown that monocyte and neutrophil expression of CD11a, CD11b and CD62L and lymphocyte CD62L increased in response to OLTT (Esser et al. 2013a, b), suggesting a generalized immune activation in response to OLTT. Subtle temporary increases in TNF-α have been observed after the OLTT challenge (Wopereis et al. 2013), but other inflammatory markers did not show differential profiles compared to a control challenge, which was in line with gene expression results in PBMC (Wopereis et al. 2013).

OLTT also induces oxidative stress in healthy subjects. Total antioxidant status levels increased from 2 to 8 h after consumption of high fat (Musso et al. 2009). In addition, increased levels of nitrotyrosine were observed 2–4 h after OLTT (Ceriello et al. 2004). These results are in line with findings in healthy obese subjects, showing increased plasma levels of oxidative stress markers TBARS and dichlorofluorescein (DCF) upon intravenous and oral fat loads (Gosmanov et al. 2010). Oxidative stress is associated with hepatocyte apoptosis, which was reflected by elevated levels of cytokeratin-18 fragments (CK-18) 6 h after OLTT in healthy subjects (Musso et al. 2009).

### OLTT response in sub-healthy and diseased conditions

Review of OLTT challenges in sub-healthy and diseased condition revealed that responses of several processes related to phenotypic flexibility, including lipid metabolism, endothelial response capacity and oxidative stress, are modified. The OLTT-induced TG response is augmented in several conditions such as overweight, obesity, T2DM and non-alcoholic steatohepatitis (NASH) (Blaak et al. 2006; Ceriello et al. 2004; Dekker et al. 2007; Harte et al. 2012; Musso et al. 2009; Nagashima and Endo 2011; Wu and Yu 2004). OLTT-induced changes in total plasma FFA, a marker for lipolysis, are affected in profile as well as in amplitude. Blaak et al. (2006) reported a blunted response in the first hour, which correlated with body mass index (BMI), whereas increased FFA responses have been shown for NASH patients, overweight subjects with elevated TG levels, subjects with MetS and obese coronary heart disease patients for the late part (>2 h) of the response curve (Dekker et al. 2007; Musso et al. 2009; Wuesten et al. 2005). Additionally, the response amplitude of FFA was larger in NASH patients compared to healthy subjects (Musso et al. 2009). In contrast to the lack of response of cholesterol-related parameters other than plasma chylomicron in healthy subjects, significant decreases in high-density lipoprotein cholesterol (HDLc) over time upon the high-fat meal were observed in subjects with overweight, impaired glucose tolerant (IGT) and T2DM (Harte et al. 2012). In another study, total cholesterol was found to slightly increase in healthy subjects but not in obese and T2DM subjects in response to a fat challenge (Umpaichitra et al. 2004).

The OLTT reveals differences in metabolic flexibility, defined as their capacity to switch from carbohydrate to lipid oxidation, between subjects with different BMI levels on OLTT (Blaak et al. 2006). Subjects with lowest BMI showed the largest increases in relative fat oxidation, accompanied by lower respiratory quotients, expressed as percentage of energy expenditure. These differences in metabolic flexibility between healthy subjects and subjects with MetS became evident only upon OLTT (Kardinaal et al. unpublished work). Different conditions of sub-optimal health also affect the amplitude of adipokine responses upon an OLTT. Leptin levels decreased in obese subjects (Gosmanov et al. 2010), while an oral lipid bolus did not affect leptin levels in T2DM patients (Wu and Yu 2004). In NASH patients, the OLTT-induced resistin increase was augmented, whereas adiponectin levels were found to decrease, in contrast to increased levels of adiponectin in healthy subjects (Musso et al. 2009). Interestingly, glucose-dependent insulinotropic polypeptide GIP showed a delayed plasma clearance in NASH subjects in response to an OLTT as compared to control subjects (Musso et al. 2009), which was recently confirmed in subjects with MetS compared to healthy subjects (Kardinaal et al. unpublished work). GIP is well known for its role in glucose metabolism by enhancing insulin secretion, but is increasingly considered to be a key modulator of lipid metabolism based on effects found in preclinical and clinical studies (Hansotia et al. 2007; Kim et al. 2007; Gault et al. 2007; McClean et al. 2007). Another incretin GLP-1 showed a significant postprandial OLTT response in subjects with MetS, in contrast to healthy subjects (Kardinaal et al. unpublished work).

Obese subjects have a less pronounced decrease in heart rate after OLTT (Esser et al. 2013a, b). So far, only minor differences in OLTT-induced effects related to endothelial response capacity have been reported between subjects with different health statuses. While the response profile of the AIX response is similar in lean, obese and T2DM subjects, the area under the curve for the AIX was higher in the T2DM patients and lean subjects compared to the obese subjects (van Dijk et al. 2012). In addition, CYP-derived oxylipins 11,12-diHETrE, 8,9-diHETrE and oxylipin 19,20-dihydroxydocosapentaenoic acid (19,20-DiHoPE) showed a blunted increase in subjects with MetS compared to healthy subjects (Kardinaal et al. unpublished work), although this difference was not confirmed in comparisons between lean and obese subjects (Esser et al. 2013a, b; Strassburg et al. 2014).

A differential response to the OLTT between healthy subjects and subjects with MetS was observed for IL-6 and IL-18. The IL-6 response was blunted, whereas an augmented IL-18 response was observed in subjects with MetS (Kardinaal et al. unpublished work). Furthermore, increased levels of complement C3 during the whole postprandial OLTT time-course were found in MetS subjects, which were not statistically different at baseline (Kardinaal et al. unpublished work). Lymphocyte CD11a and CD11b expression decreased in lean subjects after consumption of OLTT, but did not change in obese subjects (Esser et al. 2013a, b).

Besides differences in phenotypic flexibility regarding processes related to lipid metabolism, OLTT responses of other processes were also influenced by impaired health states. Despite lower maximum peak values, increases in insulin and C-peptide levels lasted longer in T2DM patients and obese males with or without cardiovascular disease compared with healthy subjects (Blaak et al. 2006; Umpaichitra et al. 2004; Wu and Yu 2004; Wuesten et al. 2005). In contrast, mean insulin levels were shown to decrease significantly after an OLTT in overweight males with elevated baseline TG levels (Dekker et al. 2007). OLTT increases endotoxin levels in several sub-optimal health conditions, i.e., obesity, IGT and T2DM, while no response was detected in healthy subjects, possibly reflecting impaired gut barrier function (Harte et al. 2012). Finally, postprandial differences in response to oxidative stress, i.e., decreased plasma total antioxidant status (TAS) concentrations, and hepatocyte apoptosis, were observed between controls and patients with NASH (Musso et al. 2009).

### The oral protein tolerance test

Since the early 1960s, protein tolerance tests have been described in various scientific publications for medical applications. However, the effect of ingesting a 100 % protein bolus on metabolic processes in healthy subjects has hardly been studied. A dose of 1.5 g/kg casein was administered to one healthy subject resulting in a temporary decrease in non-esterified fatty acid (NEFA) levels (Fernandes and van de Kamer 1968). In other studies, the protein challenges consisted of various test meals e.g., red meat, eggs, cheese, chicken, which contain large amounts of protein but also substantial amounts of fat. An increase in plasma creatinine was reported as well as an increase in glomerular filtration rate after ingesting 70–80 g of protein in the form of red meat (Bosch et al. 1984). Table 3 summarizes the physiological effects of these two types of protein challenges.

**Table 3:** Processes of phenotypic flexibility modulated by OPTT

Organ system	Process	Direction (↑, ↓, –)	Disturbed
Adipose tissue	Lipolysis	↓	
Kidney	(Re)absorption	↑	

Fernandes studied children with liver glycogenosis due to a deficiency in the debranching enzyme system (Fernandes and van de Kamer 1968). In these children, a protein bolus (2 g/kg body weight) resulted in a gradual increase in plasma glucose levels, with lactate levels remaining low, whereas hexose administration raised blood lactate levels and resulted in a strong fluctuation in glucose levels. NEFA decreased in the first 1.5 h and then increased again (Fernandes and van de Kamer 1968). It was concluded that glucose was formed from amino acids by gluconeogenesis. This report suggests that the type of protein administered did not affect the study results in view of similar responses to casein, egg white protein, and gliadin.

A protein tolerance test has also been proposed to identify individuals with sub-optimal renal function before they manifest clinically (Tiwari et al. 2005) and to diagnose urea cycle defects (Potter et al. 2001). Protein stresses the renal parenchyma and raises the glomerular filtration rate, reflecting the filtration capacity (Bosch et al. 1984).

### The combined carbohydrate, lipid and protein challenge test

**Fig. 3:**
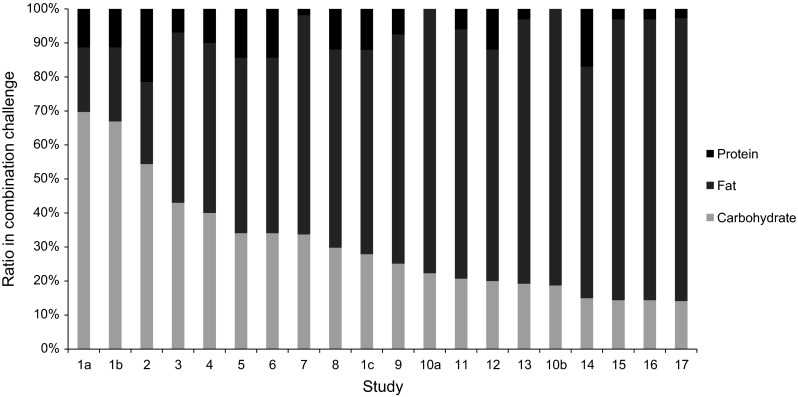
Ratio of carbohydrate, fat and protein in combination challenge tests used in reported studies. The studies are organized from high carbohydrate content on the *left* to low carbohydrate content on the *right* (*1* Esposito and Nappo, *2* Zwirska-Korczala, *3* Ramos-Roman, *4* Casas-Agustench, *5* Phillips, *6* Scheffer, *7* Wopereis, *8* Pellis, *9* Krug, *10* Thomsen, *11* Coutinho, *12* Derosa, *13* Cheng, *14* Mortensen, *15* Saxena, *16* Mahdu, *17* Iraklianou)

A total of 17 papers were identified that include aspects of phenotypic flexibility after the application of a challenge test with a combination of carbohydrates, fat and protein, i.e., an oral protein–glucose–lipid tolerance test (OPGLTT). It is assumed that an OPGLTT triggers all aspects of phenotypic flexibility as described for OGTT, OLTT and OPTT challenges. All papers looked at a healthy subject response to a combined challenge test, and ten papers described the response to or combined challenge test in various groups with a sub-optimal health status. The phenotypic flexibility responses are summarized in Table 4. The macronutrient composition of combined challenge tests is very different among the 17 papers reviewed (Fig. 3), and these compositional differences may influence the observed responses. This aspect will be addressed in a separate paragraph.

**Table 4:** Processes of phenotypic flexibility modulated by OPGLTT

Organ system	Process	Direction (↑, ↓, –)	Disturbed
Gut	Incretin production	↑	T2DM
	Satiety hormone production	↑ and ↓	Obesity
Adipose tissue	Lipolysis	↑	T2DM
	Adipokine production	↑	T2DM
Systemic stress	Insulin sensitivity	↑	T2DM, MetS, obesity
	Oxidative stress	↑	T2DM
	Inflammation	Slight ↑	T2DM, MetS
	ER stress	↑	
	Metabolic flexibility	–	Obesity
Liver	β-oxidation	↑	Obesity
	Ketogenesis	↑	
	Glycolysis/Gluconeogenesis	↑	
	Lipoprotein production	↑	T2DM, MetS
	TCA cycle	↑	
Kidney	Urea cycle	↑	
Vasculature	Blood pressure regulation	↓	T2DM
	NO formation	↓	
	Adhesion	↑	T2DM
Pancreas	Insulin secretion	↑	T2DM, obesity
Brain	Endocrine response	↓	
	Neurotransmitters and precursors	↑	

*MetS* metabolic syndrome, *T2DM* type 2 diabetes mellitus

The OPGLTT induces expected responses in plasma glucose (Ramos-Roman et al. 2012; Scheffer et al. 2011), triglyceride (Casas-Agustench et al. 2009; Esposito et al. 2003; Krug et al. 2012; Scheffer et al. 2011; Zwirska-Korczala et al. 2007) and amino acid concentrations (Pellis et al. 2012). Plasma glucose response similar to an OGTT was observed, inducing an insulin response that was prolonged in comparison with the glucose response (Casas-Agustench et al. 2009). Following protein absorption, plasma amino acid concentrations showed a rapid increase and returned to baseline within 4–5 h. Together this supports the applicability of the OPGLTT to explore phenotypic flexibility on glucose, lipid and protein metabolism.

The insulin-dependent homeostatic program along the four key metabolic axes as reported by Shaham et al. (2008) in response to an OGTT was also observed in response to OPGLTT except for the process of proteolysis. Plasma long-chain free fatty acids and glycerol concentrations increased after a lag time of 3 h (Pellis et al. 2012), demonstrating initial inhibition of the lipolysis process by insulin action followed by reactivation. FFA C10:0 rose immediately after OPGLTT, followed by a slow increase in FFA C12:0 and C14:0. Fatty acids C10:0, C12:0 and C14:0 are poor substrates for re-esterification into triglycerides, because the specific medium-chain acyl-CoA synthase required is absent. Thus, these fatty acids enter plasma directly as unesterified fatty acids (Pellis et al. 2012). The plasma concentrations of ketone bodies 3-hydroxybutanoic acid and acetoacetate coincided with the availability of free fatty acids (Pellis et al. 2012), showing that ketogenesis is also inhibited by insulin action. This is in line with the decrease in plasma acylcarnitines and increase in free carnitine to an OPGLTT, which are assumed to serve as proxies for intracellular β-oxidation intermediates (Krug et al. 2012). As a consequence of the inhibition of lipolysis, the metabolic system is focused on triglyceride storage in the adipose tissue, resulting in the reduced conversion of fatty acids into CoA thioesters that are transported via the palmitoyl-CoA carnitine transferase II shuttle into mitochondria. This acyl-CoA transport requires free carnitine, and therefore, the postprandial increase in free carnitine indicates that the cellular acylcarnitine supply is decreased by reduced free fatty acid uptake from plasma (Krug et al. 2012). The postprandial response reduction in plasma acylcarnitine is specifically seen for unsaturated species such as C14:1 and C14:2 (Ramos-Roman et al. 2012). The process of glycolysis and tricarboxylic acid (TCA) cycle was activated and recovered 3 h after the OPGLTT (Pellis et al. 2012).

The adipokines adiponectin (Esposito et al. 2003) and leptin (Iraklianou et al. 2001) decreased in response to OPGLTT. No changes in fat oxidation rate were found in 29 healthy men after three mixed meals differing in fat type where substrate oxidation was measured postprandially over a 5 h period as compared to baseline values (Casas-Agustench et al. 2009). Hepatic lipoprotein production markers related to cholesterol transport showed inconsistent responses to an OPGLTT. Some studies report increased postprandial total cholesterol and low-density lipoprotein cholesterol (LDLc) levels (Saxena et al. 2005; Scheffer et al. 2011), and decreased postprandial HDLc levels (Coutinho et al. 2008; Scheffer et al. 2011; Thomsen et al. 2003), and one study reported increased postprandial HDLc concentrations (Saxena et al. 2005). Most studies report stable LDLc (Coutinho et al. 2008; Derosa et al. 2010; Esposito et al. 2003; Iraklianou et al. 2001; Saxena et al. 2005; Wopereis et al. 2013), HDLc (Esposito et al. 2003; Iraklianou et al. 2001; Wopereis et al. 2013) and total cholesterol concentrations (Coutinho et al. 2008; Esposito et al. 2003; Thomsen et al. 2003; Wopereis et al. 2013). This possibly reflects composition-dependent differences in response.

Like the OLTT, the OPGLTT induces endothelial responses as reflected by increased concentrations of sVCAM-1 and sICAM-1 (Nappo et al. 2002; Wopereis et al. 2013). In a cross-over design study, Wopereis et al. (2013) compared three different challenge tests, OGTT, OLTT and OPGLTT, to a control challenge (Wopereis et al. 2013). This showed that endothelial function was most modified by the OPGLTT as reflected by vascular inflammatory markers, such as sVCAM-1, sICAM-1 and oxylipins associated with endothelial inflammation, and function, such as LOX-derived 9-HODE and 13-HODE and CYP-derived 11,12-DiHETrE and 14,15-DiHETrE. This suggests that the addition of glucose and protein in OPGLTT augments the endothelial response. Accordingly, postprandial levels of myeloperoxidase (MPO), and matrix metallopeptidases (MMP)-1 and MMP-9, which have been linked to impaired endothelial function, were increased by OPGLTT or a mixed meal challenge (Cheng et al. 2010; Pellis et al. 2012; Spallarossa et al. 2008).

Many studies report that OPGLTT induces temporary increase in numbers of leukocytes (Cheng et al. 2010; Coutinho et al. 2008; MacEneaney et al. 2009; van Oostrom et al. 2003, 2004; Tamburrelli et al. 2012; Wopereis et al. 2013). The increase in leukocyte numbers is primarily due to increased neutrophil numbers. This indicates that the OPGLTT induces a modest acute inflammatory response.

The assessment of inflammatory biomarker responses to an OPGLTT have generated inconsistent results (Derosa et al. 2010; Esposito et al. 2003; Nappo et al. 2002; Pellis et al. 2012; Wopereis et al. 2013). A key inflammatory cytokine, IL-6, is reported to either not respond, increase or decrease upon a challenge in healthy subjects (Derosa et al. 2010; Esposito et al. 2003; Nappo et al. 2002; Pellis et al. 2012; Wopereis et al. 2013). IL-6 levels increased similarly to nutrition stress tests as to a control challenge, suggesting that the observed effects on IL-6 are primarily a result of local tissue production of IL-6 associated with cannula placement (Wopereis et al. 2013). Interestingly, all studies that have reported effects on IL-6 after dietary challenges used a continuous intravenous line (Blackburn et al. 2006; Dekker et al. 2009; Derosa et al. 2010; Jellema et al. 2004; Lundman et al. 2007; MacEneaney et al. 2009; Poppitt et al. 2008; Tulk and Robinson 2009; Van Oostrom et al. 2003), whereas studies that have seen no effects or even decreases in postprandial IL-6 levels used venipuncture (Campbell et al. 2006) or did not report on blood sampling method (Madec et al. 2011; Manning et al. 2008; Metzig et al. 2011). Like IL-6, inflammatory markers TNF-α and C-reactive protein (CRP) showed a significant response to a water control (Wopereis et al. 2013), suggesting that reported changes in response to OPGLTT (Derosa et al. 2010; Esposito et al. 2003; Nappo et al. 2002; Pellis et al. 2012) should be reconsidered. Like for the OGTT and OLTT, the OPGLTT resulted in only minor effects on metabolic inflammation gene expression in white blood cells (Wopereis et al. 2013). Interestingly, *Il*-*18* gene expression was augmented in response to OPGLTT as compared to control challenge (Wopereis et al. 2013), which was in line with elevated plasma IL-18 concentrations upon an OPGLTT (Esposito et al. 2003), which suggest the involvement of inflammasome-related processes.

In addition, induction of an oxidative stress response by OPGLTT has been observed. Plasma TBARS increased significantly from fasting to post-meal state which peaked at 4–6 h and declined at 8 h in healthy subjects. A similar response with significant postprandial increase was observed for erythrocyte-reduced glutathione (GSH), whereas activity of superoxide dismutase was not changed (Saxena et al. 2005). Decreased levels of the antioxidant uric acid were found in 36 overweight males (Pellis et al. 2012).

Finally, endocrine metabolism and the production of gastrointestinal hormones may be modulated by OPGLTT. Testosterone, progesterone and the transport-binding protein for sex steroid hormones sex-hormone binding globulin (SHBG) showed decreased postprandial concentrations with a recovery after 6 h in 36 overweight male subjects (Pellis et al. 2012). Thyroid stimulating hormone and the thyroid hormone transporter thyroxine binding globulin (TBG) showed a similar response to OPGLTT, suggesting a temporary decreased thyroid hormone production in response to OPGLTT (Pellis et al. 2012). Also cortisol, an important metabolic hormone, counteracting the effect of insulin by binding to glucocorticoid receptors, showed a linear decrease upon OPGLTT (Pellis et al. 2012). The appetite-regulating hormones Peptide YY (PYY), cholecystokinin (CCK) and gastrin significantly increase in response to OPGLTT, whereas ghrelin and acylated ghrelin decrease in response to OPGLTT (Zwirska-Korczala et al. 2007).

### OPGLTT response in sub-healthy and diseased conditions

Differing OPGLTT responses have been reported for several processes related to phenotypic flexibility in subjects with sub-optimal health status. Increased amplitudes of glucose response were observed upon an OPGLTT in T2DM patients and MetS patients, compared to healthy subjects (Ceriello et al. 2004; Ramos-Roman et al. 2012; Saxena et al. 2005; Scheffer et al. 2011; Zwirska-Korczala et al. 2007). Accordingly, the postprandial insulin response was significantly higher as well as prolonged, in obese subjects and T2DM patients compared to lean subjects (Iraklianou et al. 2001; Phillips et al. 2010; Zwirska-Korczala et al. 2007).

Similar findings were reported for the postprandial TG response. Plasma TG levels showed increased amplitudes in T2DM patients compared to healthy subjects (Harte et al. 2012; Iraklianou et al. 2001; Madhu et al. 2005, 2008; Nappo et al. 2002). Also the duration of the response was increased, peaking at 8 h post challenge in MetS and T2DM patients compared to 4 h in healthy controls (Ceriello et al. 2004; Saxena et al. 2005; Scheffer et al. 2011). In contrast, Phillips et al. (2010) did not find any differences in TG responses between lean, obese and diabetic subjects (Phillips et al. 2010). The prolonged hypertriglyceridemia was reported to result in the formation of triglyceride-enriched HDLc and LDLc particles and a concomitant increased postprandial susceptibility of LDLc to oxidation, causing a pro-atherosclerotic lipoprotein profile in T2DM patients and in MetS, albeit less pronounced as compared to healthy subjects (Scheffer et al. 2011).

Lipolysis, for which total FFA is the main parameter, has also been reported to show differential responses upon OPGLTT in different health conditions. A blunted total FFA response was found in overweight subjects (Ramos-Roman et al. 2012) as well as in T2DM patients (Thomsen et al. 2003). The postprandial nadir, which is the time where the lowest postprandial concentrations of plasma acylcarnitine were reached, were positively associated with lean body mass, postprandial fatty acid flux and FFA concentrations and negatively correlated with insulin sensitivity and spillover of meal-derived fatty acids (Ramos-Roman et al. 2012).

OPGLTT results in a significant increased GIP and GLP-1 response as compared to a control challenge in T2DM patients (Thomsen et al. 2003). GIP and GLP-1 concentrations were not back to baseline levels in T2DM patients 8 h after the OPGLTT (Thomsen et al. 2003). The gastrointestinal hormones related to satiety that act in close harmony with the incretins also showed changes in response to OPGLTT. PYY and (acylated) ghrelin response was blunted or was absent in (morbid) obese women as compared to lean women, whereas cholecystokinin (CCK) showed a higher and prolonged response (Zwirska-Korczala et al. 2007). Gastrin showed a limited increase, but prolonged clearance from plasma in obese and morbid-obese (Zwirska-Korczala et al. 2007). Differences in these parameters were only observed by the challenge response, as plasma concentrations of PYY, acylated ghrelin, CCK and gastrin levels in the fasting state were similar between obese and lean women.

Related to endothelial response capacity, several differences between different health sub-phenotypes were reported. The time to return to baseline levels for AIX was longer in T2DM patients as compared to lean subjects (Phillips et al. 2010). The endothelial adhesion markers sE-selectin, sVCAM-1 and sICAM-1 increased in T2DM patients after ingesting a high-fat meal, but the peaks were reached after 2 h instead of 1 h as observed in healthy subjects (Ceriello et al. 2004; Nappo et al. 2002). Additionally, in T2DM patients, higher elevations of sVCAM-1 and sICAM-1 were found 4 h after the OPGLTT compared to healthy subjects (Nappo et al. 2002).

The amplitude, as well as the duration of OPGLTT-induced response of several inflammation markers, including IL-6, IL-18 and TNF-α, was reported to be augmented in T2DM patients compared to healthy subjects (Esposito et al. 2003; Nappo et al. 2002). It should be noted that many of the low-grade inflammation markers, including IL-6 and TNF-α, showed increased levels in plasma at baseline after an overnight fast in impaired health conditions.

Finally, differences have been reported in activation of an oxidative stress response by OPGLTT. The postprandial rise in TBARS levels was larger in T2DM patients with macroangiopathy than in T2DM patients without macroangiopathy and healthy subjects (Saxena et al. 2005). In contrast, GSH levels remained lower in the T2DM patients compared to healthy subjects (Saxena et al. 2005). Furthermore, in T2DM patients with higher fasting triglyceride levels also higher malondialdehyde (MDA) levels were found, which subsequently showed a more pronounced response after the challenge (Saxena et al. 2005).

### Effects of macronutrient composition and fat type on the challenge response

In the literature reviewed on OLTT and OPGLTT challenge response, several studies reported on differences related to macronutrient composition or type of fat used for the challenge test. It can be expected that differences in composition result in triggering of differential responses, which may be the cause of varying results reported in literature. Since the main aim of this review is to define the optimal composition of a standardized nutritional stress response test, the influence of macronutrient composition and fat type is summarized in this paragraph. Of the 61 papers used for this review, two papers looked at OPGLTT challenge tests that differed in their macronutrient composition (Esposito et al. 2003; Nappo et al. 2002), while seven papers described the influence of type of fat on OLTT or OPGLTT responses (Casas-Agustench et al. 2009; Dekker et al. 2007; Esser et al. 2013a, b; Newens et al. 2011; Strassburg et al. 2014; Thomsen et al. 2003; van Dijk et al. 2012). The influence of different challenge test compositions on phenotypic flexibility processes is summarized in Table 5.

**Table 5:** Overview of modulated processes for OPGLTT and OLTT challenge tests differing in macronutrient composition and fatty acid type

Organ system	Process	60 E% FAT	70 E% CAR	SFA	MFA	PUFA
Gut	Incretin production				↑	
Adipose tissue	Lipolysis			↑^b^	↑^b^	↑
	Lipokine and adipokine production	↓	–			
Systemic stress response	Systemic insulin sensitivity	↑	↑^a^		↑	
	Chronic low-grade inflammation	↑^a^	–	↑		
	Metabolic flexibility			↑	↑↑	↑↑↑
Liver	Lipoprotein production	↑^a^	–	↑^a,b^	↑^b^	↑
Vasculature	Vascular flexibility			↓↓^b^	↓↓^b^	↓
	Adhesion	↑	–	↑		
	Oxylipin response			↑	↑	↑↑

^a^Differential response between healthy and sub-optimal healthy subjects

^b^Inconsistency in literature

Esposito et al. (2003) compared effects of OPGLTT challenges that differed in carbohydrate and fat content (Fig. 3, challenge 1a/1c) on the response of parameters related to endothelial health and inflammation in healthy subjects and T2DM. They showed that a “high fat” OPGLTT (challenge 1c, 12 E% protein, 28 E% carbohydrates and 60 E% fat) induces a significant increase in triglycerides, TNF-α, IL-6, IL-18, sICAM-1 and sVCAM-1 levels, and a decrease in adiponectin levels in healthy subjects, whereas this response was absent in the “high-carb” OPGLTT (challenge 1a, based on 11 E% protein, 70 E% carbohydrates and 19 E% fat). The rise in triglyceride levels was significantly larger in diabetic subjects which was only observed after the “high-fat” OPGLTT. In these diabetics, both compositions triggered significantly augmented elevations in TNF-α, IL-6, sICAM-1 and sVCAM-1, and reduction in adiponectin, compared to healthy subjects. An enhanced IL-18 response was observed after “high-fat OPGLTT” in T2DM patients, but not after “high-carb OPGLTT” (Esposito et al. 2003). Both compositions lacked the induction of a response of glucose and insulin in healthy subjects, whereas they resulted in a significant rise of glucose and insulin in diabetic subjects. These responses were more sustained in response to the “high-carb” OPGLTT.

Besides differences in macronutrient composition, also different types of fat result in different phenotypic responses of the body. Challenge tests containing saturated fat (SFA), monounsaturated fat (MUFA) or PUFA were compared in the context of endothelial health as well as inflammation in healthy subjects (Dekker et al. 2007; Newens et al. 2011) or in subjects with different metabolic risk phenotypes (Esser et al. 2013a, b; Strassburg et al. 2014; van Dijk et al. 2012).

In healthy subjects, a SFA-based OPGLTT induced arterial stiffness and a sICAM-1 response, in contrast to a MUFA-based OPGLTT. Although both SFA- and MUFA-based OPGLTT induced an increased NEFA response, SFA resulted in a larger elevation in NEFA, which may explain the induced arterial stiffness and sICAM response that was observed in healthy subjects (Newens et al. 2011). Another study that compared the SFA-, MUFA- and PUFA-based OPGLTT effects on endothelial response capacity showed a higher postprandial rise of TG and a less pronounced reduction in NEFA after MUFA intake (Esser et al. 2013a, b; Strassburg et al. 2014; van Dijk et al. 2012). The higher postprandial rise of TG after MUFA intake compared to SFA intake is in line with observations from others (Mekki et al. 2002; Oakley et al. 1998; Peairs et al. 2011), but in contrast to the observation that in T2DM patients a butter meal (SFA-rich) and not an olive oil meal (MUFA-rich) caused a significant increase in plasma TG (Thomsen et al. 2003). Dekker et al. (2007) observed larger increases of total FA levels after a PUFA based as compared to a SFA-based OLTT in nine overweight males. Furthermore, TG levels in response to the PUFA-based OLTT reached peak levels faster than in response to SFA-based OLTT, i.e., at 3 and 6 h post challenge, respectively.

In both lean and obese subjects, MUFA-rich OLTT induced the largest decrease in AIX, followed by SFA- and PUFA-rich fat loads (Esser et al. 2013a, b). Ahuja suggested that this decrease in AIX is the result of changes in arterial stiffness caused by vasodilation (Ahuja et al. 2009). While Newens et al. (2011) showed a similar response for sICAM-1 in lean subjects, van Dijk et al. (2012) did not observe a difference in response of sICAM-1 between the tested fat compositions. sP-selectin expression, which stimulates adherence of leukocytes and platelets to endothelium, specifically responded to a SFA-rich fat load in lean and obese subjects, showing no response to MUFA or PUFA (Esser et al. 2013a, b). The postprandial circulating oxylipin concentrations also responded differentially to the different fatty acid types. SFA-rich OLTT induced LOX-mediated and linoleic acid (LA)-derived 9-HODE and 13-HODE, which lead to increased expression of TNF-α, sVCAM and sE-selectin and can therefore be associated with endothelial function and inflammation (Shearer and Newman 2009; Wang et al. 2009). MUFA-rich OLTT induced 9,10-DiHOME and decreased 12,13-DiHOME, which are CYP2J2 epoxygenase-derived oxylipins from linoleic acid, which are produced under inflammatory conditions although their biological function is poorly understood (Askari et al. 2014). Most effects on circulating oxylipins were observed after n-3 PUFA-rich OLTT, which induced increases in CYP450-mediated and AA-derived DiHETrEs and oxylipins from EPA and DHA precursors. The CYP450-derived DiHETrE’s may be released from the vascular endothelium and lead to vasodilation and vascular smooth-muscle relaxation via stimulation of Ca^2+^-activated K^+^ channels in coronary arteries or via modulation of endothelial nitric oxide (NO) release (Giordano 2011). Compared to SFA challenge, the MUFA and n-3 PUFA challenges induced larger changes in expression of inflammation genes *MCP1* and *Il1β* in PBMCs (van Dijk et al. 2012). Based on these results, van Dijk et al. (2012) suggested using MUFA-based OLTT to reveal differences in endothelial response capacity of subjects.

In addition to effects on endothelial and inflammatory response, differential effects of fatty acid types on other processes have been described. The thermogenic effect of a PUFA-rich meal was 28 % higher than of a MUFA-rich meal and 23 % higher compared to a SFA-rich meal (Casas-Agustench et al. 2009). The thermogenic effect was expressed as postprandial energy expenditure corrected for resting metabolic rate. This is in agreement with other studies that show higher postprandial fat oxidation relative to carbohydrates and higher thermogenesis after MUFA as compared to SFA meals (Piers et al. 2002; Soares et al. 2004).

In T2DM patients, HDLc levels specifically decreased upon a SFA-rich OPGLTT challenge, while showing no response after a MUFA-rich OPGLTT challenge. In contrast, total cholesterol levels did not decrease after the SFA-rich challenge, but decreased substantially after the MUFA-rich challenge. Examining the response of chylomicron production by the gut showed that a SFA-rich OPGLTT induced significantly higher TG concentrations in the chylomicron-rich fraction than did the MUFA-rich OPGLTT, while both exceeded the response to the control meal (Thomsen et al. 2003). The GLP-1 response to a MUFA-rich challenge was larger than that to the SFA-rich meal in T2DM patients, whereas a similar GIP response between the two challenges differing in fat type was observed in both healthy and T2DM subjects (Newens et al. 2011; Thomsen et al. 2003). A higher glucose and insulin response was observed after the MUFA-rich OLTT challenge as compared to SFA-rich and PUFA-rich challenges in both lean and obese (van Dijk et al. 2012), whereas in healthy subjects, a second insulin peak was observed after the MUFA-rich OPGLTT, which was absent in SFA-rich OPGLTT (Newens et al. 2011).

### Effects of nutritional interventions on the nutrition stress test response

A total of four studies have applied challenge tests in the context of nutritional interventions. An OPGLTT consisting of a 500 ml shake consisting of 300 ml custard, 150 ml cream and 50 ml whipping cream was applied to investigate effects of a 5 week intervention with an anti-inflammatory dietary mix in healthy overweight men with mildly elevated CRP levels (Pellis et al. 2012). The intervention resulted in differential challenge responses of 31 plasma parameters. After the anti-inflammatory intervention, OPGLTT challenge induced delayed and reduced increases of macrophage-derived chemokine and myeloperoxidase (52 and 25 % reduction respectively), suggesting a reduced oxidative stress response. Additionally, CD40, sVCAM-1, fibrinogen and epidermal growth factor receptor (EGF-R) showed a stronger and more rapid decrease in response to the challenge after the dietary intervention, potentially reflecting a pronounced acute vascular inflammatory response. Glucagon increased only in response to the challenge after the dietary intervention and not after the placebo intervention. Indole-3-propionic acid, which is synthesized by gut microbiota, decreased in response to the challenge after the dietary intervention in the late phase, while in the placebo situation, the levels were elevated.

In the LIPGENE study (Cruz-Teno et al. 2012), 75 subjects with MetS were randomly assigned to one of four interventions: high saturated fatty acids (HSFA), high monounsaturated fatty acids (HMUFA), low-fat high complex carbohydrate with long-chain n-3 polyunsaturated fatty acids (LFHCC n-3) and low-fat high complex carbohydrate with placebo (LFHCC). After 12 weeks of dietary intervention, the response to a high-fat challenge meal with the same fat composition as the intervention diet was measured for each of the intervention groups. Specific responses to the high-fat challenge tests were the postprandial decrease in nuclear factor NF-κB activation and nuclear p65 protein levels and increase in transcription activity of PBMC inhibitory molecule from NF-kB (*IkB*-*α*) gene in the HMUFA diet group. NF-κB regulates the expression of cytokines, chemokines and other molecules involved in inflammation. An increase in NF-κB activity is therefore considered a pro-inflammatory response which is inhibited by *IkB*-*α*. This suggests an anti-inflammatory effect of a high monounsaturated fatty acid diet in MetS patients, which was revealed by the application of a challenge test after the dietary intervention.

In the chocolate study, the effects of high flavonol chocolate (HFC) and normal dark chocolate (NFC) on vascular health was investigated in a double-blind cross-over study in healthy overweight middle-aged men (Esser et al. 2014). It was shown that chocolate consumption resulted in increased FMD, and decreased AIX, total leukocyte counts, and plasma soluble adhesion molecules in response to an OLTT. These results are in line with the substantiated health claim for flavonols, i.e., bioactive in chocolate, on maintenance of endothelium-dependent vasodilation. The lack of differences observed between HFC- and NFC-induced effects suggested that increased flavonol concentrations did not have an added beneficial effect on vascular health.

Finally, in the overfeeding study, an OLTT was applied to study the effects of 4 weeks of chronic high-fat, high-caloric (HFHC) diet (~additional 1300 kcal/day extra) in 10 healthy men (Kardinaal et al. unpublished work). As a reference, the OLTT was applied in 10 MetS subjects. The OLTT response revealed an altered postprandial response of markers related to systemic stress, vascular health and metabolic flexibility. After the HFHC intervention, several parameters, such as IL-8, GLP-1, GIP and the 19,20-DiHoPE, responded similarly as in the MetS subjects. Other modulated response parameters, specifically related to the systemic stress response, were only observed as a consequence of the intervention, indicating activated processes dealing with the chronic high calorie exposure.

## Summary and conclusions

The objective of this review was to identify a composition for a nutrition stress test that will assess phenotypic flexibility by triggering responses in all processes deemed relevant, based on detailed comparison of different challenge tests. An overview of those processes that are modulated by the different challenges is presented in Table 6. The OGTT triggers all insulin-dependent processes related to phenotypic flexibility in healthy subjects. In addition, this standardized challenge induces subtle systemic stress responses including oxidative stress and vasculature, and also bile production was modulated. Impaired health conditions are associated with modified OGTT responses for most of these processes. The OGTT would not be the first choice for studying effects related to lipid metabolism and beta-oxidation, in view of the reported inconsistency in results. The OLTT triggers a wide range of processes related to lipid metabolism, such as lipolysis, adipokine production and lipoprotein production. Furthermore, oxidative stress responses, endothelial responses, subtle inflammatory effects and hepatic stress responses have been described in healthy subjects. For most of these processes, a differential response to an OLTT has been observed in different states of health. Glucose and insulin responses have been reported in studies that applied an OLTT with small amounts of carbohydrates, illustrating the added value of combining different macronutrients. Finally, effects of OPTT challenges on lipolysis and urea cycle/kidney function have been described, although this challenge type has hardly been studied.

**Table 6:** Overview of modulated processes for OGTT, OLTT and OPGLTT

Organ system	Process	OGTT	OLTT	OPTT	OPGLTT
Gut	Host–microbe interaction				
	Defense against pathogens				
	Gastrointestinal functioning				
	Absorption				
	Intestinal integrity				
	Incretin production	↑	↑^a^		↑^a^
	Satiety hormone production				↑^a^
	Gut-mediated inflammation		–^a^		
Adipose tissue	Lipolysis	↓^a^	↑^a^	↓	↑^a^
	Adipose insulin sensitivity				
	Expandability				
	Lipokine and adipokine production	–	↑^a^		↑^a^
	Macrophage infiltration				
Systemic stress response	Systemic insulin sensitivity	↑^a^	–		↑^a^
	Oxidative stress	↓^a^	↑^a^		↑^a^
	Nitrosative stress	↑^a^	↑^a^		
	Chronic low-grade inflammation	–	–^a^		↑^a^
	Metabolic flexibility		↑^a^		–^a^
	ER stress	↓			↑
Muscle	Protein metabolism	↑^a^			
	Muscle tissue injury control	↓			
	Muscle insulin sensitivity				
Liver	β-oxidation	↓^a^			↑^a^
	Citric acid cycle				↑
	Ketogenesis	↓^a^			↑
	Glycolysis/Gluconeogenesis	↑^a^			↑
	Purine degradation	↓			
	Hepatic protein metabolism				
	Lipoprotein production	↓^a^	↑^a^		↑^a^
	Bile production	↓^a^			
	Hepatic fat metabolism				
	Fibrosis and inflammation				
	Hepatic insulin sensitivity				
	Hepatic injury control		↑^a^		
Kidney	(Re)absorption		↑		
	Urea cycle	↓			↑
Vasculature	Blood pressure regulation	↓^a^	↓^a^		↓^a^
	NO formation	↓			↓
	Vascular flexibility		↓^a^		
	Adhesion	↑^a^	↑^a^		↑^a^
	Complement system				
	Coagulation system				
Pancreas	α-Cell				
	β-Cell	↑^a^	↑^a^		↑^a^
Brain	Endocrine response				↓
	Secondary messengers				
	Mental performance				
	Appetite				
	HPA axis	–^a^			
	Neurotransmitters and precursors	↓			↑

^a^Differential response between healthy and sub-optimal healthy subjects

The concept of a mixed macronutrients challenge test, termed OPGLTT, is that this would facilitate the combined evaluation of the response of phenotypic flexibility processes triggered by each individual challenge test. In general, this was confirmed by our review of OPGLTT challenge test studies. The OPGLTT evidently triggers the lipid-related processes to a similar extent as to an OLTT, and insulin-dependent processes triggered by glucose show a clear response similar as to an OGTT. Additionally, the OPGLTT response of endothelial flexibility, oxidative stress and inflammation processes is augmented compared to the individual macronutrient challenges. Interestingly, OPGLTT consistently triggers endothelial and inflammatory responses in subjects with sub-optimal health, whereas healthy and young subjects often do not show a response of these processes. Remarkably, modulation of phenotypic flexibility of metabolic processes and processes related to vascular health, inflammation and oxidative stress by nutritional interventions could only be shown by application of an OPGLTT. Although information on challenge responses of wide range of processes is lacking, the insights gathered suggest that to trigger the widest array of phenotypic flexibility-related processes, the challenge test should ideally consist of glucose, lipids and protein.

Comparative studies revealed that compositional differences in fat species and macronutrient ratios are of significant influence on OPGLTT- and OLTT-induced responses. This review revealed the high variability in composition of these challenge types used in phenotypic flexibility studies (Fig. 3), which makes it hard to compare results. This illustrates the need for harmonization and standardization of challenge tests, preferably consisting of glucose, lipids and protein. The insights from this review do, however, provide guidance for proposing an optimal composition for a standardized OPGLTT. A minimum amount of 35 % glucose is required to obtain a response in glucose and insulin metabolism and related physiological processes. The required percentage protein cannot be determined based on the available literature, although it suggests the importance of protein in studying gluconeogenesis and renal function. A minimum of 30 % fat is required to obtain a response in chylomicron and VLDL-TG production and processes related to lipid metabolism, although at least 30 % fat is needed in to induce a HDLc response. The FA species, i.e., MUFA, PUFA and SFA, all have their specific response characteristics. MUFA particularly triggers a response in blood pressure regulation and incretin GLP-1 production. SFA induces a specific strong response in chylomicron production and TG and cholesterol metabolism. Finally, PUFA induces a specific strong thermic response as well as a particularly strong response in adipose tissue lipolysis. In conclusion, we propose that a standardized OPGLTT for evaluating effects on phenotypic flexibility should contain at least 35 % glucose, 30 % fat and protein. Our aim is to develop a standardized challenge test that will be implemented as the golden standard for measuring health effects of the consumption of functional ingredients and food. In this context, we are testing a nutritional stress test with 75 g of glucose, 60 g palm olein and 20 g protein, in the form of a 400 ml water-based drink. Palm olein is a blend of MUFA, SFA and PUFA (36.6 % MUFA, 48.8 % SFA, 9.1 % PUFA and 5.5 % other/unknown fatty acids) in healthy and diseased populations. To achieve further standardization of the challenge test, we provide study subjects with a standardized meal the night before the nutritional stress test to limit the potential introduction of variation due to acute dietary intake. Finally, based on the insights from this review, we suggest adopting a standardized design of studies aiming to establish health effects of diet or nutritional components. Optimally, these studies have a placebo-controlled randomized cross-over design, establishing challenge responses after each study period. If the cross-over design is not achievable, studies should at least comprise a placebo control arm, and challenge responses should be determined before and after the treatment period, to establish specific modulation of the challenge response by the dietary intervention.

This review identified a significant knowledge gap related to the impact of health-modifying factors on challenge responses. Due to a wide variety of reasons, such as genetic background, gender, age, (micro)nutritional status, habitual food intake, psychological stress and physical activity, individuals differ in their “wiring” of phenotypic flexibility and therefore will react differently to acute and chronic stressors and develop a personal trajectory of metabolic-inflammatory health and disease (van Ommen et al. 2014). The nutritional stress test could help to better quantify personal health by revealing the “weak spots” in the phenotypic flexibility network and health effects from diets. It is adamant to characterize how it modulates the different processes of phenotypic flexibility shown in Fig. 1 and how well it differentiates between health states in the sequel from optimal healthy to sub-optimal healthy toward diseased, including heterogeneity, subpopulations and different stages of the disease. A key factor is determining the physiological relevance of specific changes in phenotypic flexibility in a healthy (sub)population, in response to a dietary intervention. Ideally, this is pursued by validation against established markers of health, but these do not exist. Alternatively, modifications in challenge responses can be correlated to established markers of disease or to long-term health outcomes (disease risk, longevity) in cohort studies for validation purposes. Multi-biomarker panels will emerge that act as composite descriptors of physiological processes. In the example of vascular health, such a composite marker could be composed of FMD, a functional marker of endothelial function and blood pressure, resilience markers for endothelial damage after a metabolic challenge test, such as sVCAM, sICAM and sE-selectin response, total cholesterol and specific single nucleotide polymorphisms related to increased risk of cardio-metabolic disease development. By combining this into an integrated readout, a flexibility marker for vascular health can be obtained that has a broader value, both for substantiating the effects of food and nutrition on health, but also for application in personalized healthcare.

In conclusion, the application of a challenge test allows the evaluation of subtle differences in health status, as well as the evaluation of subtle effects of nutrition on health. Particularly, in the area of nutritional research, subtle effects of diet and dietary components are expected in subjects with sub-optimal health and early state of impaired health and also in healthy subjects. Even though a limited number of studies have been reported, challenge tests allow the detection of early and subtle changes in phenotypic flexibility as a result of nutritional intervention. This review provides further evidence for the applicability of nutrition stress tests to assess phenotypic flexibility and paves the way for future nutrition health research.

## Electronic supplementary material

Supplementary material 1 (PDF 44 kb)

Supplementary material 2 (XLSX 48 kb)
