# Supplementary material for: Phenotypic flexibility as a measure of health: the optimal nutritional stress response test
Source: Genes Nutr. 2015 Apr 21;10(3):13. doi: 10.1007/s12263-015-0459-1 (PMC4404421; doi:10.1007/s12263-015-0459-1)
Supplement: Supplementary file 1 — Supplementary material 1 (PDF 44 kb) [file 12263_2015_459_MOESM1_ESM.pdf]

Online Resource 1

Phenotypic Flexibility as a measure of health: the optimal nutritional stress response test for quantification

Johanna H.M. Stroeve*, Herman van Wietmarschen*, Bas H. A. Kremer, Ben van Ommen, Suzan Wopereis

TNO, Zeist, the Netherlands

* Both authors contributed equally to this work

Corresponding author and request for reprints: Suzan Wopereis, PO Box 360, 3700 AJ Zeist, tel: (+31) 088 8665013, suzan.wopereis@tno.nl

Online Resource 1. Health markers related to phenotypic flexibility markers

Organ system	*Process*	*Plasma Markers*	*Other measurements*
Gut	Host-microbe interaction	acetate<br>proprionate<br>butyrate<br>indole-3-propionic acid (IPA)	metabolomics in faeces<br>metagenomics in faeces
	Absorption	lactoluse<br>mannitol<br>campesterol<br>sitosterol	
	Intestinal integrity, barrier function	xylose<br>arabinose<br>ribose<br>fructose<br>maltose	
	Gut hormone production	GLP-1<br>GIP<br>ghrelin<br>Acylated ghrelin<br>CCK<br>Gastrin<br>Motilin<br>PYY	
	Gut-mediated inflammation control	LPS in plasma<br>Endotoxin<br>IgA	

	Chylomicron production		lipoproteins
Brain	Endocrine responses	Orexin<br>Amylin<br>Cortisol<br>testosterone<br>progesterone<br>Luteinizing hormone (LH)<br>sex hormone binding globulin (SHBG)<br>endocannabinoids<br>CRH<br>ACTH	
	Secondary messengers	Inositol<br>Myoinositol	
	HPA axis		heart rate variability
	Neurotransmitters & precursors	Norepinephrine<br>Epinephrine<br>Histamine<br>Histidine<br>Dopamine<br>Tyrosine<br>Tryptophane<br>Serotonine<br>Aspartate<br>Glutamate<br>GABA<br>Glycine<br>Acetylcholine<br>Phenylalanine	

		Methionine<br>Thyroid stimulating hormone (TSH)<br>thyroxine binding globulin (TBG)	
Adipose tissue	Lipolysis & Lipotoxicity	NEFA<br>FFA<br>Monoglycerides<br>ceramides<br>diacylglycerols<br>glycerol<br>LPL activity	
	Adipose insulin sensitivity	NEFAxfasting insulin<br>oxylipids	
	Expandability		body composition measurements (Inbody, DEXA, MRI)<br>estimated SCD activity in plasma
	Lipokine & Adipokine production	adiponectin<br>C16:1n7<br>leptin<br>resistin	enzyme activity measurements in adipose tissue biopsies
	Macrophage infiltration		Crown like structure staining in adipose tissue biopsies

			cytokine and chemokine measurements in adipose tissue biopsies
Systemic stress response	Systemic insulin sensitivity	HbA1C<br>HOMA-IR<br>Glucose<br>1,5-anhydroglucitol	Matsuda index
	Oxidative stress	ROS<br>uric acid<br>myeloperoxidase (MPO)<br>Malondialdehyde<br>DCF<br>TBARS<br>methionine sulfoxide<br>Superoxide dismutase (SOD) activity<br>total antioxidant status<br>vitamin E<br>hydroxy fatty acids<br>Conjugated dienes<br>Glutathione balance<br>F2-isoprostanen<br>8-OH-dG in urine (=8-hydroxydeoxyguanosine; an oxidized nucleoside of DNA)<br>PG-isoF2-alpha in urine	Comet assay

	chronic low-grade inflammation	IL-1β<br>IL-6<br>IL-8<br>IL-10<br>IL-12p70<br>IL-18<br>TNF-α<br>IFN-γ<br>CD40<br>MCP1<br>CXCL9<br>CXCL10<br>IL1Ra<br>MIP1-β/CCL4<br>EN-RAGE/S100A12<br>MDC/CCL22<br>n-3/n-6 ratio<br>oxylipids	FACS analysis<br>haematology<br>NFkB activity in blood cells<br>PLA2 activity<br>plasma challenge test response, resolvins and protectins
	adaptation carb/lipid switch		Energy expenditure<br>fat oxidation<br>Respiratory quotient<br>carbohydrate oxidation
	ER stress	mannose	
Muscle	Protein metabolism	isoleucine<br>leucine<br>valine<br>3-methyloxovaleric acid	

		4-methyl-2-oxovaleric acid	
	Muscle tissue injury control	creatine<br>Lactate<br>1-methylhistidine<br>3-methylhistidine<br>4-hydroxyproline<br>methyl-hydroxyproline<br>hydroxylysine<br>beta-alanine<br>sarcosine<br>carnitine<br>carnosine<br>anserine<br>3-nitrotyrosine<br>myoglobin<br>creatine kinase	
	Muscle Insulin sensitivity		Muscle IR index
Liver	Core metabolism	methionine<br>ribose<br>hypoxanthine<br>glycine<br>pseudo uridine	β-oxidation<br>citric acid cycle<br>ketogenesis<br>PPP<br>glycolysis/glyconeogenesis
	Lipoprotein production	VLDL<br>HDL<br>total cholesterol<br>free cholesterol<br>other sterols<br>SPM, PC, LPC, ChE<br>LDL	

		TG<br>apolipoprotein C-III<br>apolipoprotein A-1	
	Bile production	bile acids	
	Hepatic tissue injury control	GGT<br>ALP<br>ASAT<br>ALAT<br>CK18 fragments<br>Bilirubin	
	Fibrosis & inflammation	CRP<br>TIMP<br>tenascin C	
	Hepatic insulin sensitivity	Liver IR index<br>Liver IS index	
Kidney	(re)absorption	creatinine<br>cysteine	
	urea cycle	aspartic acid<br>glutamic acid<br>ornitine<br>urea<br>albumin	
Vasculature	Blood pressure regulation		Systolic/Diastolic BP<br>Heart rate (BPM)<br>kinin system markers in plasma<br>Endothelium-independent<br>Augmentation Index (AIX)<br>vasodilatation by nitroglycerin

	Endothelial integrity & flexibility	nitrite/nitrate<br>arginine<br>citrulline<br>SDMA<br>ADMA<br>SAA<br>fibrinogen<br>E-selectin<br>P-selectin<br>VCAM1<br>ICAM1<br>ICAM3<br>vWF<br>PAI-1<br>complement 3<br>serum amyloid P<br>thrombomodulin<br>factor VII<br>apolipoprotein H<br>MMP-1<br>MMP-2<br>MMP-9<br>glycosylated hemoglobin<br>homocysteine<br>Endothelin-1<br>Angiotensin-converting enzyme (ACE)	MRI, echoing, imaging, scanning
Pancreas	alpha-cell function	glucagon	
	beta-cell function	PPY	

		insulin<br>C-peptide<br>pancreatic polypeptide - PP<br>Disposition index<br>HOMA-B		
